# Validation of an IMU Suit for Military-Based Tasks

**DOI:** 10.3390/s20154280

**Published:** 2020-07-31

**Authors:** Matthew P. Mavor, Gwyneth B. Ross, Allison L. Clouthier, Thomas Karakolis, Ryan B. Graham

**Affiliations:** 1 Faculty of Health Sciences, School of Human Kinetics, University of Ottawa, Ottawa, ON K1N 6N5, Canada; mmavo062@uottawa.ca (M.P.M.); gwyneth.ross@uottawa.ca (G.B.R.); aclouthi@uottawa.ca (A.L.C.); 2 Defence Research and Development Canada, Government of Canada, Toronto, ON M3K 2C9, Canada; Thomas.Karakolis@drdc-rddc.gc.ca

**Keywords:** inertial sensors, Xsens, army, joint kinematics, principal component analysis, PCA, root mean squared error, RMSE, Xsens vs. Vicon

## Abstract

Investigating the effects of load carriage on military soldiers using optical motion capture is challenging. However, inertial measurement units (IMUs) provide a promising alternative. Our purpose was to compare optical motion capture with an Xsens IMU system in terms of movement reconstruction using principal component analysis (PCA) using correlation coefficients and joint kinematics using root mean squared error (RMSE). Eighteen civilians performed military-type movements while their motion was recorded using both optical and IMU-based systems. Tasks included walking, running, and transitioning between running, kneeling, and prone positions. PCA was applied to both the optical and virtual IMU markers, and the correlations between the principal component (PC) scores were assessed. Full-body joint angles were calculated and compared using RMSE between optical markers, IMU data, and virtual markers generated from IMU data with and without coordinate system alignment. There was good agreement in movement reconstruction using PCA; the average correlation coefficient was 0.81 ± 0.14. RMSE values between the optical markers and IMU data for flexion-extension were less than 9°, and 15° for the lower and upper limbs, respectively, across all tasks. The underlying biomechanical model and associated coordinate systems appear to influence RMSE values the most. The IMU system appears appropriate for capturing and reconstructing full-body motion variability for military-based movements.

## 1. Introduction

Load carriage is an important component of typical occupational activities for military soldiers. When in the battlefield and during training, soldiers wear bulky clothing, stiff armour, and carry heavy backpacks. The items a soldier carries are lifesaving and operationally-relevant. However, they may also lead to musculoskeletal injuries over time [1,2,3] and impair overall performance (e.g., movement speed and exposure time) [4,5]. To better understand the demands placed on the body by typical military loads, researchers have studied the effects of load carriage on joint kinematics using optical motion capture. Operationally-relevant loads increase trunk flexion, lower limb flexion range of motion (ROM), and walking speed during gait [6,7]. However, optical motion capture is generally confined to use in a laboratory setting and measuring motion in a more natural working environment is not typically feasible. Furthermore, optical motion capture requires line of sight between the cameras and body-mounted markers, which makes it challenging to study the effects of wearing military equipment as the markers must not be covered.

Inertial measurement units (IMUs) are becoming increasingly popular as a method of measuring human movement that overcomes some of the barriers of optical motion capture. IMUs are small devices that contain sensors to make inertia-based measurements of motion. These may consist of an accelerometer, gyroscope, and magnetometer that measure linear acceleration, angular velocity, and variations in the magnetic field, respectively. A range of kinematic and kinetic variables, including joint angles can be calculated by fusing data from these sensors and incorporating constraints from a biomechanical model [8,9]. IMUs can be placed directly on the skin, can be covered with clothing, and are highly transportable, which directly addresses the aforementioned limitations of optical systems. These benefits of IMU technology allow researchers to study individuals in their natural environment, while wearing the appropriate clothing and equipment needed to perform a given task.

Growing interest in the potential of IMUs has led to a number of validation studies that aim to assess the accuracy of joint kinematics calculated using these wearable sensors. Typically, accuracy has been quantified by comparing joint angles calculated using IMU systems to optical motion capture through root mean squared error (RMSE) [10,11], correlation coefficients [12,13], and/or Bland-Altman limits of agreement [14,15]. Efforts have largely been focused on IMU system validation for lower limb angles during gait [16,17,18]. However, other activities, including stair climbing, kicking, materials handling, and skiing [14,19,20,21] and upper body angles [22,23] have been examined as well. Lower limb sagittal plane angles have been found to have relatively low RMSE ranging from 2–11° [24,25,26,27] and good correlation coefficients from 0.9–1.0 [11,12,28] with optically-derived angles. Errors tend to be greater and correlations worse for frontal and axial plane angles [12,19,25]. Studies reporting full-body angles have demonstrated larger errors for upper body angles, compared to lower limb angles [14,29]. These previous IMU system validation studies were aimed at investigating movements that target the lower limbs and manual materials handling tasks where there was little translation by the participant. These are not representative of the prone positions or rapid changes in height that are typically performed by soldiers. As a result, the validity of using an IMU system to measure military-based movements remains unclear.

Our larger, overarching goal is to generate morphable models of movement using principal component analysis (PCA) and linear discriminant analysis. That is, we intend to incrementally alter and represent military movement patterns between two body-borne load conditions without actually collecting an intermediate load. This will allow us to simulate a spectrum of body-borne load conditions that are infeasible to collect. Before this can be accomplished, we must establish an accurate method of measuring full-body movement during typical military activities in the field while wearing operationally-relevant loads. The purpose of this investigation was to compare an IMU system (MVN BIOMECH Link, Xsens, Enschede, the Netherlands) with an optical (OPT) motion capture system (Vantage V5, Vicon, Oxford, UK) for a variety of operationally-relevant military movements. To be comparable to previous research, we compared the RMSE of the joint angles calculated using the IMU system to the OPT system. We calculated joint angles using the IMU data in multiple ways to determine the differences between systems that could be expected in practice, and to examine any difference due to the biomechanical models used by each system and the technology itself. We also examined the agreement of the systems in terms of full-body movement variability as captured using PCA as this pattern recognition technique will be used in the future to generate morphable models of movement.

## 2. Materials and Methods

### 2.1. Participants

Ten male and 10 female civilian participants were recruited for this study. However, due to technical difficulties caused by both systems, which made the data unusable, only nine males and nine females were analyzed. Mean age, height, and mass of all participants were 23.7 years (standard deviation (SD) = 3.44), 175 cm (SD = 7.93), and 71.9 kg (SD = 13.2), respectively; all participants’ demographics are reported in Table 1. All participants read and signed the participant information letter, which was approved by the University of Ottawa ethics board (approval number H-06-18-721), before data collection began.

### 2.2. Participant Preparation and Equipment

Participants were asked to change into athletic shorts and a Lycra^®^ T-shirt that is specifically designed for the Xsens system. The T-shirt has Velcro^®^ locations on the shoulders to place sensors, zippers on the back to contain wires, and pockets in the back to host the battery and onboard computer.

Participants were first outfitted with 17 IMU sensors, which were placed on the back of the head, sacrum, sternum, and bilaterally on the upper arms, forearms, hands, shoulders, thighs, shanks, and feet. The pelvis, upper limb, and lower limb sensors were affixed to the skin using neoprene bands, specialty gloves were used for the hand sensors, an elastic headband was used for the head sensor, the trunk sensors (sternum and shoulders) were placed in the T-shirt described above, and the feet sensors were placed inside participants’ shoes using a neoprene insert.

A cluster-based optical marker set was then placed atop the IMU system. Passive four-marker clusters were affixed to the body with Velcro^®^ atop the IMU system’s neoprene bands to be in a similar locality as the IMU system for each segment. The four-marker cluster placed approximately over the T_10_–T_12_ vertebrae was placed using a neoprene band that wrapped around the abdomen and over the IMU system’s battery pack and onboard computer; the cluster was placed between the hardware. A three-marker cluster was taped onto the dorsal aspect of the toe bed of the participants’ shoes and four individual markers were taped onto the headband. Individual markers were also taped to specific anatomical landmarks (bilaterally on the medial and lateral malleoli, medial and lateral condyles of the humerus and femur, greater trochanter of the femur, radial and ulnar styloid processes, PSIS, ASIS, iliac crests, acromions, and dorsum of the hands and on C7) so a whole-body biomechanical model could be created.

**Figure 1:**
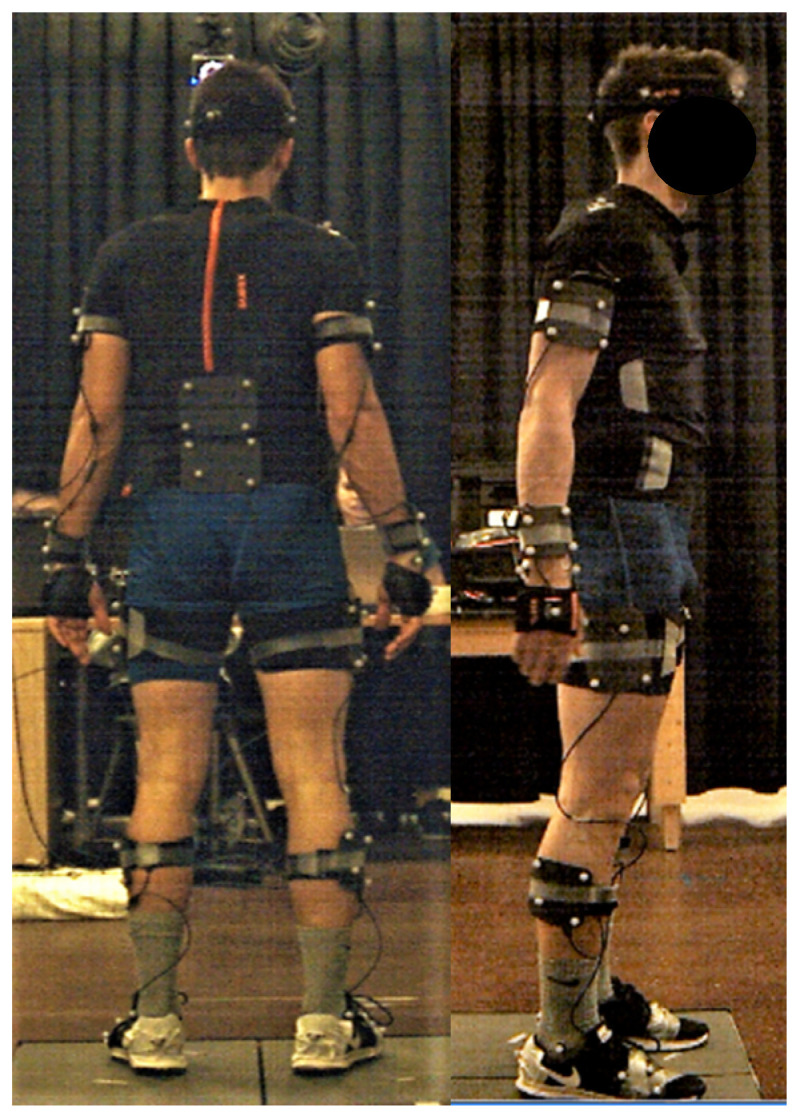
Participant setup. A cluster-based optical marker set was worn on top of the Xsens IMU suit.

Participants’ kinematic data were captured simultaneously from both the IMU (MVN BIOMECH, Xsens, Enschede, the Netherlands) and OPT (Vantage 5, Vicon, Oxford, UK) motion capture systems at 240 Hz. A common event of hand clapping/floor smacking was used to synchronize both motion capture systems. A visual representation of the participant setup is displayed in Figure 1.

### 2.3. Movement Protocol

Participants were asked to perform eight operationally-relevant military movements: running, walking, kneel-to-prone (KTP), prone-to-kneel (PTK), kneel-to-run (KTR), prone-to-run (PTR), run-to-kneel (RTK), and run-to-prone (RTP). All tasks were performed three times in a straight line with the exception of walking, where participants were asked to walk in a criss-cross pattern measuring 3.5 m × 2 m twice (i.e., two repetitions of walking forward 3.5 m, turning 135° clockwise to walk on a diagonal 4 m, turning 135° counter clockwise to walk forward 3.5 m, and turning 135° counter clockwise to walk on a 4 m diagonal to return to the start position).

Due to the limited field of view of the OPT system, participants started ~3.5 m outside of the capture volume for the RTK and RTP movements and were instructed to transition into kneeling or prone in the middle of the capture volume. Conversely, during the KTR and PTR movements, participants started in the middle of the capture volume and were instructed to run ~3.5 m outside the capture volume. For the running movement, participants started ~3.5 m outside the capture volume and were instructed to run through the capture volume, beginning their deceleration ~3 m past the capture volume. For movements that began outside the capture volume, participants performed the hand clap in the middle of the OPT capture volume, jogged to the start position, performed the movement, and then hand clapped/floor smacked at the end of the movement. For those movements ending outside of the OPT capture volume, participants completed the movement then jogged back to the middle of the capture volume to perform a hand clap.

### 2.4. Data Processing

**Figure 2:**
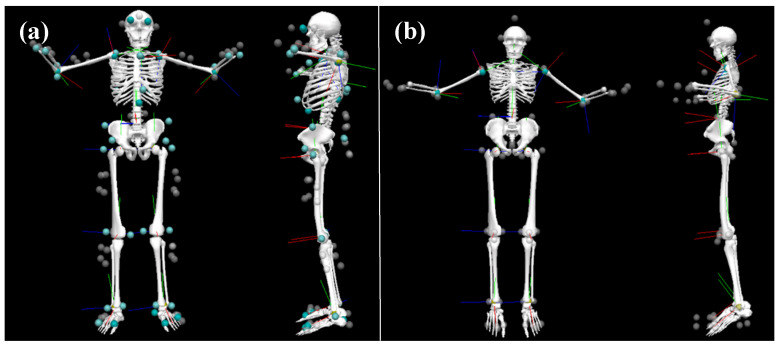
Example Visual3D model for a representative participant. Visual3D’s 6 degree-of-freedom model was used for all joint angle calculations. (**a**) Model built from OPT data (V_OPT_). (**b**) Model built with IMU data (V_IMU_). Tracking markers used can be found in Supplementary Material Table S2.

Three-dimensional (3D) time series marker trajectories from all trials and repetitions were exported from the OPT motion capture system to coordinate 3D file format (C3D). Prior to exporting, OPT marker trajectories were gap filled and filtered using a 4th order Butterworth filter in Nexus 2.5 (Vicon, Oxford, UK). MVN Analyze (Xsens, Enschede, The Netherlands) provides 64 3D time series marker trajectories of virtual markers for the IMU data that are generated based on the pose of each segment and their underlying biomechanical model. These were exported in C3D format as well. For PCA, only those markers that were similar between systems (N = 30; Supplementary Material Table S1) were imported into Matlab (2018b The MathWorks, Natick, MA, USA). For the joint angle RMSE analysis, C3D data from both systems (Supplementary Material Table S2) were imported into Visual3D (C-motion, Germantown, MD, USA) to calculate joint angles using the Visual3D six degrees of freedom pose computation algorithm (Figure 2), which were subsequently imported into Matlab for further analysis (V_OPT_ and V_IMU_). Joint angles were calculated a second time from the Visual3D IMU data following the application of a method to better align the segment anatomical coordinate systems to those in the optical data model (V_IMU-CAL_). In this procedure, for each body segment the angular velocities of adjacent segments were calculated with respect to that body segment for both V_OPT_ and V_IMU_ models throughout all motion trials. This was used to calculate a transformation matrix between the anatomical coordinate systems of the two models for each body segment. The transformation matrix was applied to the coordinate systems in the V_IMU_ model to calculate V_IMU-CAL_ [30]. This enabled a closer comparison of the two measurement technologies by mitigating error due to coordinate system alignment [14]. Joint angles were also calculated directly from the IMU data in the MVN Analyze software and imported into Matlab for comparison (X_IMU_). All data were synchronized using the peak acceleration of the hand markers (OPT)/hand segments (X_IMU_) during the hand clap/floor smack events.

#### 2.4.1. Principal Component Analysis

For both systems, 3D marker trajectories were synchronized then cropped. For the walk and run trials, each gait cycle was cropped based on heel strike, while the remaining movement trials were cropped based on visually-identified start and end points. All trials were then normalized to 101 data samples using a piecewise cubic Hermite interpolating polynomial (PCHIP). These data were reshaped from the original 101 × 90 matrix (101 data points × (30 markers * 3 axes)) to a 1 × 9090 vector. For each movement type, the newly shaped vectors were horizontally concatenated to create a N × 9090 matrix, where the first $\frac{N}{2}$ rows were OPT data and the last $\frac{N}{2}$ rows were IMU data. PCA was applied to each N × 9090 matrix.

PCA was applied separately for each movement based on the way the participants performed the movement. The KTP, PTK, PTR, KTR, RTP, RTK, and Run tasks were separated as left or right movers. For example, for the KTP task, the participants who kneeled with their left leg were considered left movers versus participants who kneeled with their right leg were right movers, and, therefore, were analyzed through PCA separately. This right versus left kneeling criteria was used for the prone transitional tasks as well, as a kneel is a functional movement in the transition into and out of prone. Run was separated based on either a left or a right gait cycle. Additionally, for the walking task, gait cycles were separated by left and right sides as well as the four directions that made up the crisscross pattern (i.e., left gait and right cycles were analyzed separately for the initial forward progression, both diagonals, and the second forward progression for a total of 8 PCA models). Movements were analyzed separately based on left and right sides because PCA identifies the greatest modes of variance within a dataset. Therefore, if both left and right movers were included in the same analysis, PCA would identify left versus right as the greatest difference, rather than the variance within the time series marker trajectories, which is of much greater interest.

For each movement type, the number of principal components (PCs) retained was the minimum amount to describe >90% of the variance within the marker trajectories. The PC scores for each retained PC were compared between the OPT and IMU systems using Pearson correlations; Spearman rank correlations were used for those data that violated parametric assumptions. Correlation coefficients from left and right sides as well as each walking direction were averaged together to represent one correlation coefficient for each retained PC for each movement. Correlation coefficients were interpreted as negligible (0.00 to ± 0.30), low (± 0.30 to ± 0.50), moderate (± 0.50 to ± 0.70), high (± 0.70 to ± 0.90), and very high (± 0.90 to ± 1.00); direction of the correlation was interpreted as either positive or negative. Variance explained by each PC from left and right sides were averaged as well.

#### 2.4.2. Root Mean Squared Error

Joint angles calculated through Visual3D and MVN Analyze were imported into Matlab 2018b where a bias for each joint angle was removed by subtracting the joint angles from a standing calibration trial that was shared by both systems. Data were synchronized then cropped and normalized to 101 data samples using PCHIP, as described above. Following normalization, the value of the first frame was subtracted throughout the entire joint angle so that all systems began at 0°. To ensure synchronization, flexion-extension data were compared using cross-correlation for all joints between V_OPT_ and V_IMU_, V_OPT_ and V_IMU-CAL_, and V_OPT_ and X_IMU_. Those trials that had an optimal lag greater than 10 frames or less than −10 frames were excluded. For each joint, RMSE was calculated four times: (1) OPT calculated though Visual3D and IMU calculated through MVN Analyze (V_OPT_ vs. X_IMU_), (2) both systems calculated through Visual3D (V_OPT_ vs. V_IMU_), (3) both systems calculated through Visual3D with the segment coordinate systems alignment procedure for the IMU data (V_OPT_ vs. V_IMU-CAL_), and (4) IMU data calculated by both MVN Analyze and Visual3D with coordinate system alignment (X_IMU_ vs. V_IMU-CAL_) for each movement trial. V_OPT_ versus X_IMU_ was included to assess the overall difference between systems. V_OPT_ vs. V_IMU_ compares differences between the systems when the IMU joint angles are calculated in a way that is more similar to the method used for the OPT data while not requiring any information from the OPT system. V_OPT_ vs. V_IMU-CAL_ was included to examine the difference due to technology while mitigating errors arising from differences in segment coordinate system alignment. X_IMU_ vs. V_IMUCAL_ was included to examine differences arising between models while controlling for segment coordinate system alignment between MVN Analyze and Visual 3D with no effect of technology.

## 3. Results

### 3.1. Principal Component Analysis

**Figure 3:**
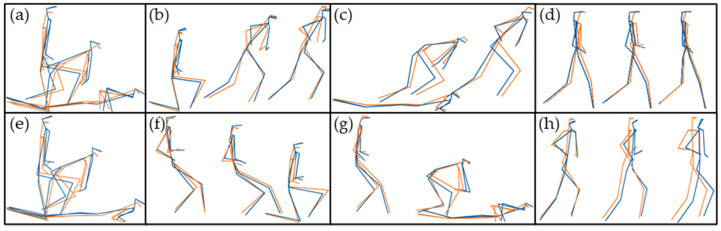
Military Movements. **Blue** represents an average OPT mover; **orange** represents average IMU mover (**a**) Kneel-to-prone (KTP); (**b**) Kneel-to-run (KTR); (**c**) Prone-to-run (PTR); (**d**) Walking; (**e**) Prone-to-kneel (PTK); (**f**) Run-to-kneel (RTK); (**g**) Run-to-prone (RTP); (**h**) Running.

Between 4 and 9 PCs were retained for each movement to explain >90% of the variance for a total of 48 PCs across all movement types. Of the 48 retained PCs, 38 (79%) had scores with a high or very high positive correlation (≥ +0.70) between the OPT and IMU systems, 15 (31%) of which had scores with a very high correlation (≥ +0.90). All correlation coefficients can be found in Table 2. A visual representation of the average movers obtained by the IMU and OPT systems are in Figure 3.

### 3.2. Root Mean Squared Error

**Figure 4:**
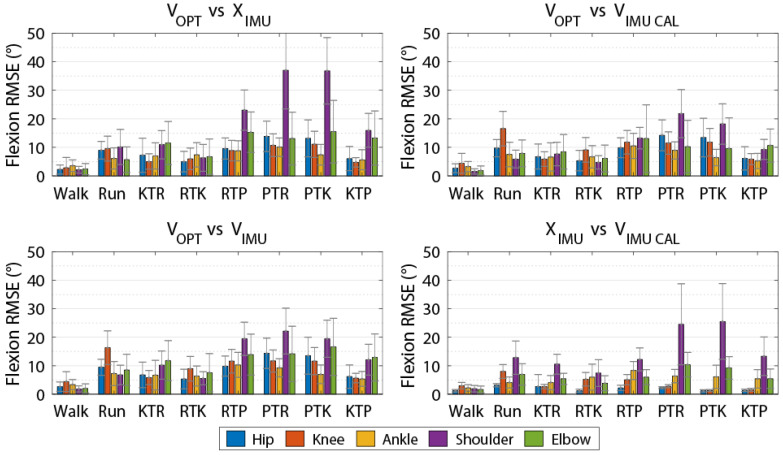
Joint flexion/extension angle RMSE values for each task for joint angles calculated from optical data using Visual3D (V_OPT_), IMU data using Visual3D (V_IMU_), IMU data using Visual3D with calibration to align anatomical coordinate systems with optical model (V_IMU-CAL_), and IMU data using MVN Analyze (X_IMU_). Presented data are averaged between left and right sides across all participants and repetitions for each trial. RTK = run-to-kneel; RTP = run-to-prone; KTR = kneel-to-run; PTR = prone-to-run; KTP = kneel-to-prone; PTK = prone-to-kneel.

The magnitude of differences between kinematics calculated using the four methods varied depending on the type of movement performed (Figure 4). RMSE was lowest for walking trials, with RMSE values less than 5° for all axes and joints for OPT compared to IMU data processed in Visual3D, with and without the additional coordinate system alignment procedure (V_OPT_ vs. V_IMU_ and V_OPT_ vs. V_IMU-CAL_). RMSE was less than 5° for all axes and joints for V_OPT_ vs. X_IMU_ with the exception of the left elbow axial rotation, which was less than 10°. Errors were greater for the other tasks, which involved faster movements and larger ranges of motion.

Comparing OPT angles calculated in Visual3D with IMU angles calculated in MVN Analyze (V_OPT_ vs. X_IMU_; Table 3) for all tasks, the mean RMSE across all joints was 10.2° (SD = 4.27) for flexion/extension, 9.30° (SD = 5.54) for ab/adduction, and 17.8° (SD = 15.7) for axial rotation. RMSE values for this comparison were typically greater than the other comparisons. Axial rotation for the upper limb differed considerably between the two methods, with a mean RMSE of 35.6° (SD = 25.1).

Comparing both OPT and IMU joint angles calculated through Visual3D (V_OPT_ vs. V_IMU_; Table 4) for all tasks, the mean RMSE across all joints was 9.61° (SD = 2.04) for flexion/extension, 7.28° (SD = 3.06) for ab/adduction, and 9.33° (SD = 4.84) for axial rotation. RMSE was less for lower limb joints than upper limb joints.

For the V_OPT_ vs. V_IMU-CAL_ comparison (Table 5), the mean RMSE across all joints for all tasks was 8.74° (SD = 1.25) for flexion/extension, 5.42° (SD = 1.52) for ab/adduction, and 7.18° (SD = 2.69) for axial rotation. The procedure to better align the anatomical coordinate systems in the IMU data with those of the OPT biomechanical model reduced the RMSE values compared to V_OPT_ vs. V_IMU_, as expected.

Calculating joint angles from the IMU data in MVN Analyze versus Visual3D with the coordinate system alignment procedure (X_IMU_ vs. V_IMU-CAL_; Table 6) resulted in mean RMSE across all joints for all tasks of 6.09° (SD = 4.31) for the flexion/extension axis, 7.45° (SD = 7.91) for ab/adduction, and 11.9° (SD = 13.2) for axial rotation. While errors in lower limb angles were generally relatively small, the different biomechanical models used to calculate the angles resulted in large difference in the upper limb joint angles, especially in the ab/adduction and axial rotation axes.

**Figure 5:**
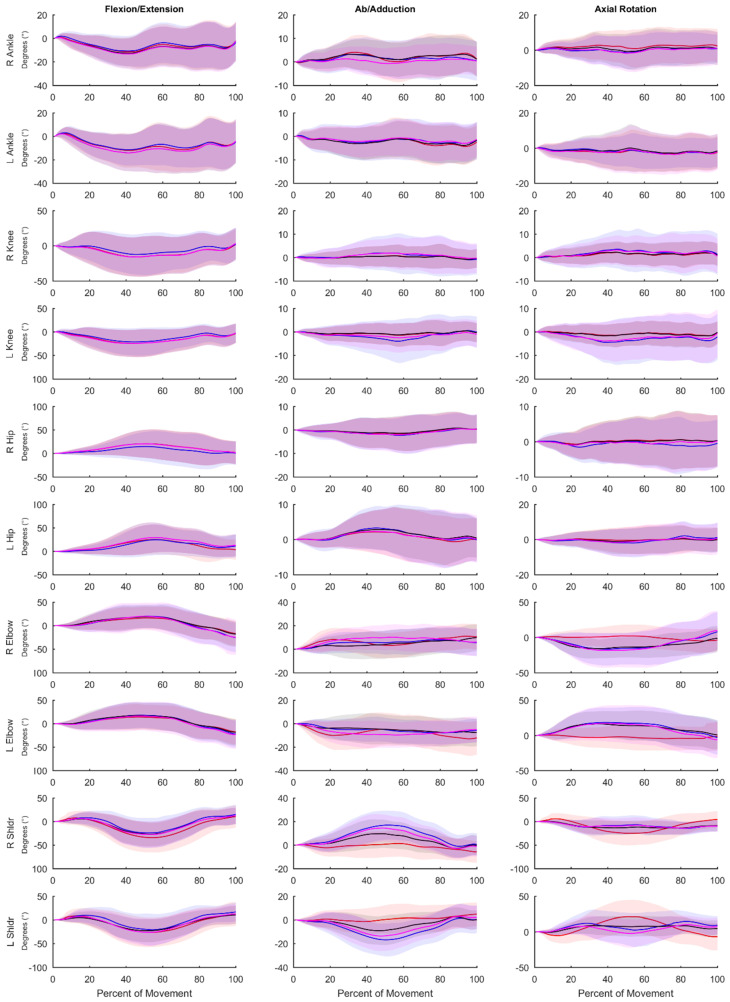
Average waveforms across all movements. **Blue** represents data captured through OPT (V_OPT_); **red** represents data captured through IMU (X_IMU_); **black** represents IMU data processed through Visual3D (V_IMU_); **magenta** represents IMU data processed through Visual3D with the coordinate system alignment procedure (V_IMU-CAL_). Shaded areas represent one standard deviation from the mean trajectory and are displayed in the same colour.

Visual representations of the average waveforms across all movements for V_IMU_, V_IMU-CAL_, X_IMU_, and V_OPT_ can be found in Figure 5.

## 4. Discussion

The purpose of this investigation was to validate an IMU system against a gold standard OPT system for military-based movements using PCA and joint angle RMSE. Overall, the OPT and IMU systems produced PC scores that were highly positively correlated; the average correlation coefficient was 0.81 (SD = 0.14) across all 48 retained PCs (Table 2). This result instills confidence that both the OPT and IMU systems are reconstructing whole-body movement patterns similarly. Lower limb RMSEs for joint angles calculated using the IMU data for all methods compared to the OPT system were less than 10 degrees for all axes (Table 3, Table 4, Table 5 and Table 6), while differences were greater between the systems for the shoulder and elbow angles. The angles measured using the OPT and IMU systems were most similar for walking trials, and RMSE tended to be greater for more rapid motions involving larger ranges of motion. RMSE was generally smaller for lower limb joint angles compared to upper limb angles.

Previous validation research of the Xsens IMU system against a gold standard OPT system during over ground walking [11,19,25] and stair ascent [19] reported lower limb joint angle RMSE values less than 5° [25], 4° [19], and 6° [11] in the flexion axis and less than 8° [19,25] and 10° [11] for the other axes. In the current study, for over ground walking, we observed joint angle RMSE values of less than 5° for the lower limbs across all axes when comparing V_OPT_ vs. X_IMU_. Across all tasks studied, we observed V_OPT_ vs. X_IMU_ RMSE values of less than 8° for the lower limbs across all axes. Our results are also comparable to [14] who reported RMSE values less than 8° for the lower limbs and shoulder axial rotation RMSE up to 40° during a manual materials handling task versus the 31.5° reported here. Robert-Lachaine et al. [14] did report smaller differences for the knee (flexion RMSE 3.2° vs. 7.3° presented here) and elbow (axial rotation RMSE 12.2° vs. 39.9° presented here) compared to the present study. However, the higher RMSE values presented here may be attributed in part to the nature of the movements studied (i.e., material handling vs. rapid changes in height and large ranges of motion).

We used multiple methods in calculating joint angles from the IMU data to provide some insight into the sources of differences between the IMU and OPT systems. The V_OPT_ vs. X_IMU_ comparison is what would be expected during a typical analysis session, where overall differences are a result of coordinate system alignment, biomechanical model constraints, and the measurement technology used between the two systems. We found that the overall RMSEs between V_OPT_ vs. X_IMU_ were less than 9° for the lower limbs but up to 40.5° for the upper limbs. The V_OPT_ vs. V_IMU_ comparison was included to investigate the differences between the systems using a similar underlying biomechanical model for calculating joint angles, which resulted in an average reduction in RMSE values for the upper limb angles of 9.3° (21.6° vs. 12.3°) across all axes compared to the V_OPT_ vs. X_IMU_ comparison, while slightly increasing the error in the lower limb angles (<2.5° for knee flexion and <0.5° for all others). To further mitigate the error associated with coordinate system definition and focus on differences due to technology alone, we used an alignment procedure based on segment angular velocities [14,30]. The V_OPT_ vs. V_IMU-CAL_ comparison further reduced the RMSE between the OPT and IMU systems, especially in the upper limbs where RMSE values decreased on average by 13.0° (21.6° vs. 8.65°) across all axes compared to V_OPT_ vs. X_IMU_. Robert-Lachaine et al. [14] used this method previously and found differences between OPT and IMU systems due to measurement technology alone to be less than 4° for most angles. While, we observed RMSEs of similar magnitude during walking, across all tasks, RMSEs were close to 10° for many of the angles studied. Finally, we examined RMSE arising from model differences, while controlling for coordinate system alignment between systems (X_IMU_ vs. V_IMU-CAL_). We observed that model differences contributed to a significant portion of the error, with RMSE values for the elbows up to 33.2°, while lower limb RMSE values were lower, indicating the lower limb model constraints and structure were more closely aligned between the two systems. However, model differences even when a coordinate system alignment procedure is applied made a large contribution to the overall error between the systems. Overall, the differences in the model and the constraints therein appear to influence RMSE values to a greater extent than the technology itself as whole-body ab/adduction and axial rotation, as well as upper limb flexion RMSE values were considerably lower in the V_OPT_ vs. V_IMU-CAL_ (system error with coordinate system alignment) than in the X_IMU_ vs. V_IMU-CAL_ comparison (model difference while controlling for coordinate system alignment).

Typical use of the Xsens system (V_OPT_ vs. X_IMU_) results in differences in joint angles and time series marker trajectories compared to an OPT system. However, these differences are relatively low for the lower limbs (RMSE of less than 8° across all axes) and whole-body time series marker trajectories have similar modes of variance between systems (average correlation coefficient of 0.81 across the 48 retained PCs). Upper limb joint angle RMSE values are quite high (RMSE of 21.6° across all axes). However, if upper limb joint angles are of great interest, these values can be decreased by mitigating differences in biomechanical models with (V_OPT_ vs. V_IMU-CAL_; RMSE of 8.7° across all axes) or without (V_OPT_ vs. V_IMU_; RMSE of 12.3° across all axes) a coordinate alignment procedure. The coordinate system alignment procedure requires simultaneous collection of optical motion capture, which may negate some of the advantages of using an IMU system. However, the results of this comparison demonstrate that, particularly in the upper limbs, a large portion of the differences between the systems (V_OPT_ vs. X_IMU_) is a result of the biomechanical model, as opposed to differences in the actual measured position and orientation of body segments. Strictly looking at time series marker trajectories, PCA identified similar modes of variance between OPT data and virtual markers projected by the IMU system, with high or very high (≥0.70) PC score correlation coefficients in 38 of the 48 comparisons. This shows that time series marker trajectories can be reconstructed similarly between data derived from the Xsens IMU and the Vicon OPT systems. The RMSE values (average of 8.7° across all joints, axes, and tasks for V_OPT_ vs. V_IMU_) provide an indication of the differences, compared to an OPT system that could be expected if the reconstructed markers are to be used for joint angle calculations. Whether this is acceptable will depend on the application. However, it indicates that joint angles similar to that of an OPT system can be calculated without the inherent line of sight and laboratory constrictions. The presented RMSE values in addition to the highly correlated PC score values show that the Xsens system is appropriate for our future purposes of creating morphable movement patterns. Users interested in other applications should be aware of the presented errors and make an informed decision if they are within an appropriate range for their intended purposes.

The present study has limitations. Although we have used the OPT system as a ‘gold standard’ for comparison, there are also errors associated with these systems. Therefore, the RMSEs we have calculated represent the observed differences between the two systems, as opposed to the difference between the IMU system and the ‘true’ movement. Due to the limited capture volume of the OPT system, for some movements participants were instructed to start/end their movement outside of the calibrated capture volume. Entering and exiting the calibrated space may cause higher sources of measurement error in the OPT system. Additionally, all OPT systems are limited by skin artefact, which would alter joint angle calculations, especially for more dynamic movements. IMU systems, when collecting for long periods of time, experience positional drift. It was also noted that upper arm segments would become misaligned over time (i.e., when the participants touched their hands together one hand of the Xsens avatar would go through the other). Although, the Xsens avatar was realigned between movement trails, positional and segmental drift could contribute to lower correlation coefficients for some PCs and contribute to the higher RMSE values for the upper arm joints. Additionally, we realized some large anecdotal improvements with the Xsens tracking after discussing with their technology team who suggested: (1) a hand clap during the walking calibration process to improve upper limb tracking, and (2) to start and end all collections with the participant in standing. Unfortunately, this information was learned after this data collection ceased so it could not be implemented. However, we believe that these would improve the PC score correlation coefficients and reduce RMSE values in future work.

## 5. Conclusions

Overall, the IMU and OPT systems reconstructed the military-based movements in a similar fashion, represented by an average high positive correlation coefficient of 0.81 across the 48 retained PCs. RMSE values were the lowest in all axes for the lower limb joint angles and highest in the upper limb joint angles. The differences between the systems were due, in part, to the different biomechanical models used. While joint angles were most similar between the systems for over ground walking, the two technologies compared favourably for more dynamic movements as well. The IMU system compares well with the OPT system and appears appropriate for capturing and reconstructing full-body motion variability for military-based movements. Users interested in other applications should be aware of the presented errors and if they lie within an appropriate range for their intended purposes.

## Figures and Tables

**Table 1:** Participant demographics.

	Number of Participants	Age (years)	Height (cm)	Mass (kg)
		Mean (SD; Range)	Mean (SD; Range)	Mean (SD; Range)
All	20	23.7 (3.44; 13.0)	175 (7.93; 30.3)	71.9 (13.2; 42.2)
Female	10	22.3 (2.26; 4.00)	171 (9.23; 26.2)	63.5 (6.80; 11.3)
Male	10	25.1 (3.93; 13.0)	179 (3.91; 11.7)	80.3 (12.8; 36.3)

Note: SD = Standard deviation. Range = maximum value minus the minimum value.

**Table 2:** Retained principal component (PC) correlation coefficients. Explained variance is in parentheses (%).

Task	PC1 (%)	PC2 (%)	PC3 (%)	PC4 (%)	PC5 (%)	PC6 (%)	PC7 (%)	PC8 (%)	PC9 (%)
RTK	0.948 (75.5)	0.770 (10.1)	0.744 (4.24)	0.939 (3.46)	-	-	-	-	-
RTP	0.937 (71.5)	0.701 (13.7)	0.810 (5.72)	0.988 (3.28)	-	-	-	-	-
KTR	0.903 (50.2)	0.702 (13.6)	0.792 (9.27)	0.970 (6.78)	0.923 (4.45)	0.849 (3.13)	0.586 (2.59)	0.612 (2.13)	0.744 (1.92)
PTR	0.829 (39.7)	0.774 (27.6)	0.678 (11.6)	0.865 (4.54)	0.882 (3.21)	0.735 (2.74)	0.887 (2.85)	-	-
KTP	0.963 (48.7)	0.961 (17.6)	0.936 (10.9)	0.884 (5.10)	0.509 (4.14)	0.702 (2.84)	0.923 (2.05)	-	-
PTK	0.867 (48.7)	0.931 (27.0)	0.962 (7.80)	0.643 (4.54)	0.710 (2.97)	0.954 (2.30)	0.848 (1.83)	-	-
Run	0.541 (61.3)	0.616 (27.3)	0.874 (2.97)	0.864 (2.58)	-	-	-	-	-
Walk	0.819 (60.0)	0.485 (22.8)	0.924 (13.7)	0.867 (6.69)	0.635 (3.95)	0.644 (1.60)	-	-	-

Note: RTK = run-to-kneel; RTP = run-to-prone; KTR = kneel-to-run; PTR = prone-to-run; KTP = kneel-to-prone; PTK = prone-to-kneel.

**Table 3:** Mean RMSE values V_OPT_ vs. X_IMU_.

	Root Mean Squared Error (°)		
Joint	Flexion-Extension Mean (SD)	Ab/Adduction Mean (SD)	Axial Rotation Mean (SD)
Right Ankle	6.59 (1.76)	6.67 (1.37)	7.16 (2.58)
Left Ankle	7.34 (2.22)	6.11 (1.14)	5.90 (1.84)
Right Knee	7.52 (3.20)	4.73 (1.28)	6.44 (1.95)
Left Knee	7.15 (3.03)	4.97 (2.08)	7.62 (3.12)
Right Hip	8.07 (4.24)	3.95 (1.20)	3.87 (1.04)
Left Hip	8.38 (3.82)	4.07 (1.37)	4.22 (1.44)
Right Shoulder	19.1 (15.0)	15.2 (8.75)	31.0 (26.0)
Left Shoulder	16.5 (11.73)	15.6 (8.51)	31.9 (25.2)
Right Elbow	10.9 (5.30)	14.7 (7.00)	40.5 (27.6)
Left Elbow	10.1 (4.84)	17.1 (8.79)	39.2 (21.4)
Overall Mean	10.2 (4.27)	9.30 (5.54)	17.8 (15.7)

Note: Mean RMSE is calculated across all movement tasks. SD = standard deviation.

**Table 4:** Mean RMSE values V_OPT_ vs. V_IMU_.

	Root Mean Squared Error (°)		
Joint	Flexion-Extension	Ab/Adduction	Axial Rotation
Right Ankle	6.50 (1.99)	6.21 (1.42)	6.21 (2.18)
Left Ankle	7.35 (2.37)	5.46 (0.83)	5.93 (1.74)
Right Knee	9.83 (4.51)	4.90 (1.29)	6.52 (1.84)
Left Knee	9.17 (3.63)	5.17 (2.13)	7.77 (2.99)
Right Hip	8.43 (4.29)	4.23 (1.14)	4.03 (1.04)
Left Hip	8.57 (3.89)	4.18 (1.35)	4.35 (1.43)
Right Shoulder	11.0 (6.46)	8.79 (3.16)	11.5 (6.62)
Left Shoulder	13.4 (8.44)	10.6 (3.52)	15.1 (10.9)
Right Elbow	10.6 (4.45)	11.8 (4.06)	16.7 (7.25)
Left Elbow	11.3 (5.36)	11.5 (3.85)	15.2 (6.72)
Overall Mean	9.61 (2.04)	7.28 (3.06)	9.33 (4.84)

Note: Mean RMSE is calculated across all movement tasks. SD = standard deviation.

**Table 5:** Mean RMSE values V_OPT_ vs. V_IMU-CAL_.

	Root Mean Squared Error (°)		
Joint	Flexion-Extension	Ab/Adduction	Axial Rotation
Right Ankle	6.50 (2.09)	5.68 (1.35)	5.98 (2.13)
Left Ankle	7.23 (2.41)	5.34 (1.00)	5.90 (1.69)
Right Knee	9.91 (4.62)	4.89 (1.42)	5.38 (1.23)
Left Knee	9.30 (3.65)	4.57 (1.20)	6.07 (1.82)
Right Hip	8.36 (4.11)	3.72 (0.89)	3.97 (1.17)
Left Hip	8.64 (4.00)	4.09 (0.99)	4.06 (1.43)
Right Shoulder	9.86 (6.39)	4.67 (2.04)	8.64 (3.94)
Left Shoulder	10.6 (7.52)	5.21 (2.20)	11.0 (7.68)
Right Elbow	8.40 (3.77)	7.29 (3.46)	10.5 (3.83)
Left Elbow	8.60 (3.49)	8.73 (4.34)	10.3 (4.46)
Overall Mean	8.74 (1.25)	5.42 (1.52)	7.18 (2.69)

Note: Mean RMSE is calculated across all movement tasks. SD = standard deviation.

**Table 6:** Mean RMSE values X_IMU_ vs. V_IMU-CAL_.

	Root Mean Squared Error (°)		
Joint	Flexion-Extension	Ab/Adduction	Axial Rotation
Right Ankle	4.44 (1.43)	4.03 (1.27)	4.17 (2.00)
Left Ankle	6.17 (2.40)	3.85 (1.43)	4.33 (1.86)
Right Knee	3.80 (2.49)	0.84 (0.28)	1.12 (0.52)
Left Knee	3.58 (2.03)	0.78 (0.23)	1.22 (0.59)
Right Hip	1.83 (0.72)	0.73 (0.23)	0.79 (0.23)
Left Hip	1.95 (0.87)	0.67 (0.16)	0.85 (0.27)
Right Shoulder	15.2 (10.1)	12.1 (6.10)	22.8 (22.4)
Left Shoulder	11.9 (5.97)	11.1 (5.58)	21.1 (16.4)
Right Elbow	5.36 (2.52)	19.7 (9.86)	29.7 (23.2)
Left Elbow	6.75 (3.10)	20.7 (9.87)	33.2 (20.0)
Overall Mean	6.09 (4.31)	7.45 (7.91)	11.9 (13.2)

Note: Mean RMSE is calculated across all movement tasks. SD = standard deviation.

## Supplementary Materials

The following are available online at https://www.mdpi.com/1424-8220/20/15/4280/s1, Table S1: OPT and Xsens time series marker trajectories used for PCA, Table S2: OPT and Xsens tracking markers used for Visual 3D biomechanical model.
