# Supplementary material for: Validation of an IMU Suit for Military-Based Tasks
Source: Sensors (Basel). 2020 Jul 31;20(15):4280. doi: 10.3390/s20154280 (PMC7435666; doi:10.3390/s20154280)
Supplement: Supplementary file 1 [file sensors-20-04280-s001.pdf]

sensors

MDPI

Supplementary Material 1

Table S1. OPT and Xsens time series marker trajectories used for PCA.

Segment	OPT Marker	Xsens Marker
Head	Back of head	pBackOfHead
	Right side of head	pRightAuricularis
	Left side of head	pLeftAuricularis
Torso	Sternal Notch	pIJ
	Xyphoid process	pPX
	C7	pC7SpinalProcess
Left Upper Arm	Left acromion	pLeftAcromion
	Left lateral epicondyle of Humerus	pLeftArmLatEpicondyle
	Left medial epicondyle of Humerus	pLeftArmMedEpicondyle
Left Forearm	Left radial styloid process	pLeftRadialStyloid
	Left ulnar styloid process	pLeftUlnarStyloid
Right Upper Arm	Right acromion	pRightAcromion
	Right lateral epicondyle of Humerus	pRightArmLatEpicondyle
	Right medial epicondyle of Humerus	pRightArmMedEpicondyle
Right Forearm	Right radial styloid process	pRightRadialStyloid
	Right ulnar styloid process	pRightUlnarStyloid
Pelvis	Left ASIS	pLeftASI
	Left iliac crest	pLeftCSI
	Right ASIS	pRightASI
	Right iliac crest	pRightCSI
Left Thigh	Left greater trochanter	pLeftGreaterTrochanter
	Left lateral epicondyle of the femur	pLeftKneeLatEpicondyle
	Left medial epicondyle of the femur	pLeftKneeMedEpicondyle
Left Shank	Left lateral malleolus	pLeftLatMalleolus
	Left medial malleolus	pLeftMedMalleolus
Right Thigh	Right greater trochanter	pRightGreaterTrochanter
	Right lateral epicondyle of the femur	pRightKneeLatEpicondyle
	Right medial epicondyle of the femur	pRightKneeMedEpicondyle
Right Shank	Right lateral malleolus	pRightLatMalleolus
	Right medial malleolus	pRightMedMalleolus

Table S2. OPT and Xsens tracking markers used for Visual 3D biomechanical model.

Segment	OPT Tracking markers	Xsens Tracking Markers
Head	Back of head<br>Front of head<br>Left side of head<br>Right side of head	pTopOfHead<br>pRightAuricularis<br>pLeftAuricularis<br>pBackOfHead
Left Upper Arm	4 marker cluster placed over the lateral side of the left humerus	pLeftAcromion<br>pLeftArmLatEpicondyle<br>pLeftArmMedEpicondyle
Left Forearm	4 marker cluster placed over the dorsum of the left radius and ulna	pLeftArmLatEpicondyle<br>pLeftArmMedEpicondyle<br>pLeftUlnarStyloid<br>pLeftRadialStyloid
Right Upper Arm	4 marker cluster placed over the lateral side of the right humerus	pRightAcromion<br>pRightArmLatEpicondyle<br>pRightArmMedEpicondyle
Right Forearm	4 marker cluster placed over the dorsum of the right radius and ulna	pRightArmLatEpicondyle<br>pRightArmMedEpicondyle<br>pRightUlnarStyloid<br>pRightRadialStyloid
Thorax/Ab	4 marker cluster placed over the $T_{10} – T_{12}$ vertebrea	pPX<br>pIJ<br>pC7SpinalProcess<br>pT12SpinalProcess<br>pT4SpinalProcess<br>pT8SpinalProcess<br>pL3SpinalProcess<br>pL5SpinalProcess
Pelvis	4 marker cluster placed over the sacrum	pHipOrigin<br>pLeftASI<br>pLeftCSI<br>pLeftIschialTub<br>pRightASI<br>pRightCSI<br>pRightIschialTub<br>pSacrum
Left Thigh	4 marker cluster placed over the lateral side of the left femur	pLeftGreaterTrochanter<br>pLeftKneeLatEpicondyle<br>pLeftKneeMedEpicondyle<br>pLeftPatella
Left Shank	4 marker cluster placed over the lateral side of the left fibula	pLeftLatMalleolus<br>pLeftMedMalleolus<br>pLeftTibialTub
Left Foot	Left heel<br>3 marker cluster placed over the dorsum of the left foot	pLeftFifthMetatarsal<br>pLeftFirstMetatarsal<br>pLeftHeelCenter<br>pLeftHeelFoot<br>pLeftPivotFoot<br>pLeftToe
Right Thigh	4 marker cluster placed over the lateral side of the right femur	pRightGreaterTrochanter<br>pRightKneeLatEpicondyle<br>pRightKneeMedEpicondyle<br>pRightPatella
Right Shank	4 marker cluster placed over the lateral side of the right fibula	pRightLatMalleolus<br>pRightMedMalleolus<br>pRightTibialTub

Right Foot	Right heel<br>3 marker cluster placed over the dorsum of the right foot	pRightFifthMetatarsal<br>pRightFirstMetatarsal<br>pRightHeelCenter<br>pRightHeelFoot<br>pRightPivotFoot<br>pRightToe
